# Inhibition of protein kinase C by isojacareubin suppresses hepatocellular carcinoma metastasis and induces apoptosis *in vitro* and *in vivo*

**DOI:** 10.1038/srep12889

**Published:** 2015-08-06

**Authors:** Xing Yuan, Hao Chen, Xia Li, Ming Dai, Huawu Zeng, Lei Shan, Qingyan Sun, Weidong Zhang

**Affiliations:** 1 Department of Phytochemistry, School of Pharmacy, Second Military Medical University, Shanghai 200433, China; 2 Department of Organic Chemistry, School of Pharmacy, Second Military Medical University, Shanghai 200433, China; 3 Shanghai Institute of Pharmaceutical Industry, Shanghai 200040, China

## Abstract

Targeted inhibition of protein kinase C (PKC) inhibits hepatocellular carcinoma (HCC) proliferation and metastasis. We previously reported the cytotoxicity of a series of synthetic phenyl-substituted polyoxygenated xanthone derivatives against human HCC. In the current study, the most potent natural product, isojacareubin (ISJ), was synthesized, and its cellular-level antihepatoma activities were evaluated. ISJ significantly inhibited cell proliferation and was highly selective for HCC cells in comparison to nonmalignant QSG-7701 hepatocytes. Moreover, ISJ exhibited pro-apoptotic effects on HepG2 hepatoma cells, as well as impaired HepG2 cell migration and invasion. Furthermore, ISJ was a potent inhibitor of PKC, with differential actions against various PKC isotypes. ISJ selectively inhibited the expression of aPKC (PKCζ) in the cytosol and the translocation of cytosolic PKCζ to membrane site. ISJ also directly interacted with cPKC (PKCα) and nPKC (PKCδ, PKCε and PKCμ) and thereby inhibited the early response of major MAPK phosphorylation and the late response of HCC cell invasion and proliferation. In a hepatoma xenograft model, ISJ pretreatment resulted in significant antihepatoma activity *in vivo*. These findings identify ISJ as a promising lead compound for the development of new antihepatoma agents and may guide the search for additional selective PKC inhibitors.

Human hepatocellular carcinoma (HCC) is one of the most common solid epithelial malignant neoplasms worldwide^1^. Recently, the RAF inhibitor sorafenib was shown to improve overall survival by approximately three months in patients with advanced HCC^2^, although various adverse events have been frequently and concomitantly observed during sorafenib therapy^3^. Thus, novel therapeutic targets and chemotherapeutic agents are urgently needed.

The protein kinase C (PKC) family of serine/threonine kinases can be divided into three groups: conventional PKCs (cPKCs α, βI, βII, γ), novel PKCs (nPKCs δ, ε, η, θ, μ), and atypical PKCs (aPKCs ζ, λ/i)^4^. All members of this family bear an N-terminal regulatory domain and a C-terminal catalytic domain^5^. Both cPKC and nPKC isozymes, which contain similar C1 membrane targeting domains, are activated through membrane recruitment and allosteric binding by diacylglycerol (DAG)/phorbol esters^6^. The aPKC isozymes differ from other PKC family members and are regulated by protein interactions^7^. Upon activation, PKC isozymes are translocated to distinct cell compartments where they phosphorylate their respective substrates, such as those in the Raf/MEK/ERK MAPK cascade^8^. PKC isoforms are involved in many cellular processes^9^,^10^, and PKC overexpression is correlated with the progression and prognosis of malignant diseases, including HCC^11^,^12^,^13^. Preventing PKC translocation from the cytoplasm to the plasma membrane inhibits the phosphorylation of specific substrates and subsequent responses initiated by PKC activation. Thus, PKC antagonism may offer a unique therapeutic approach to treat HCC.

Plant-derived natural products, as well as semisynthetic and totally synthetic analogs, represent a productive source of antitumor agents. In particular, xanthones interact with several biological targets and exhibit many pharmacological activities, including antioxidant^14^, antidiabetic^15^, and anti-inflammatory effects^16^. These compounds inhibit proliferation in multiple human tumor types^17^,^18^,^19^, including HCC^20^,^21^,^22^. and may act, at least in part, by interacting with PKC, as their downstream effects are compatible with PKC inhibition. Prenylated xanthones are potent inhibitors of eukaryotic PKC *in vitro*^23^, and norathyriol reduces the PMA-induced respiratory burst and homotypic aggregation, the mechanism for which has been attributed to direct suppression of PKC activity^24^. Furthermore, the PKC modulatory activities of twenty xanthone derivatives were evaluated using an *in vivo* yeast phenotypic assay, and the tested xanthones differed in efficacy and potency towards individual PKC isoforms^25^. Therefore, xanthone derivatives may represent an important family of potent and selective PKC inhibitors for HCC therapy.

Isojacareubin (ISJ) is a natural product that contains a xanthone scaffold. This compound can be isolated from *Hypericum sarothranol*^26^, *Hypericum roeperanum*^27^, the root of *Garcinia nigrolineata*^28^, and *Garcinia xipshuanbannaensis*^29^. ISJ was initially characterized as a potent antibacterial compound against methicillin-resistant *Staphylococcus aureus* (MRSA)^30^. In addition, Han *et al.* found that ISJ inhibited the growth of HeLa-C3 cells^29^. Our group previously isolated ISJ from *Hypericum japonicum Thunb. ex Murray*^31^, and screened its biological activity^32^. ISJ was found to be active against various cancer cells, including hepatic carcinoma HepG2 cells. We report here the synthesis of ISJ and the results of antiproliferative activity screening. ISJ and its intermediate compound **7** (7,9-dihydroxy-2,2-diphenyl-6H-[1,3]dioxolane[4,5-c]xanthen-6h-one) demonstrated greater cytotoxic activity against HCC. In addition, ISJ and compound **7** exhibited pro-apoptotic effects towards HepG2 cells and impaired HepG2 cell migration and invasion. Incubation of HepG2 cell lysates with micromolar concentrations of ISJ or compound **7** decreased PKC activity. ISJ showed stronger antihepatoma potency and PKC inhibition activity *in vitro* than compound **7**. Mechanistic investigations identified PKC as the molecular target of ISJ, with differential actions against different PKC isotypes. ISJ selectively inhibited the expression of aPKC (PKCζ) in the cytosol and the translocation of cytosolic aPKC (PKCζ) to the cell membrane. ISJ also directly interacted with cPKC (PKCα) and nPKC (PKCδ, PKCε and PKCμ), leading to inhibition of the early response of major MAPK phosphorylation and the late response of HCC cell invasion and proliferation. Moreover, ISJ exhibited potent *in vivo* antihepatoma activity in a hepatoma xenograft model. Thus, polyoxygenated xanthone-based small molecule inhibitors represent promising candidates for antihepatoma drug development and may guide the search for additional selective PKC inhibitors.

## Results

### ISJ synthesis

**Figure 1:**
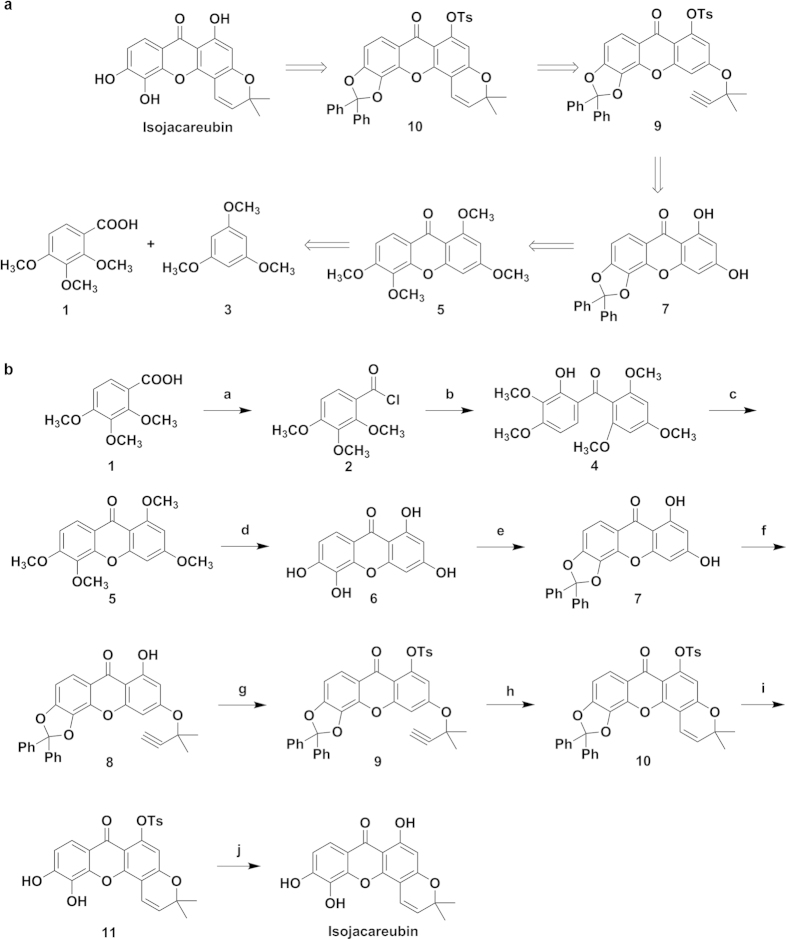
ISJ synthesis. (**a**) Retrosynthetic analysis of ISJ. (**b**) Synthetic route for ISJ. Reagents and conditions: (**a**) SOCl_2_, 80 °C, reflux, 4 h; (**b**) 1,3,5-trimethoxybenzene (compound **3**), AlCl_3_, Et_2_O, room temperature, 2 days; (**c**) pyridine, 10% (C_4_H_9_)_4_NOH, 110 °C, 36 h; (**d**) HBr, HAc, reflux, 4 days; (**e**) Ph_2_CCl_2_, DPE, 178 °C, 30 min; (**f**) 3-chloro-3-methylbut-1-yne, CuI, KI, K_2_CO_3_, acetone, argon, 45 °C-0 °C; (**g**) TsCl, K_2_CO_3_, acetone, reflux, 90 min; (**h**) DMF, 150 °C, 2 h; (**i**) CAS, THF/MeOH, reflux, 6 h; (**j**) KOH, EtOH/H_2_O, reflux, 1 h.

For ISJ synthesis, the construction of the xanthone nucleus and the benzopyran ring were performed based on our previous work^33^,^34^. The retrosynthetic analysis of ISJ is outlined in Fig. 1a. With this approach, the important intermediate, compound **7**, was synthesized in five steps from the commercially available starting materials, benzoic acid (compound **1**), with an approximately 45% yield. Then, an efficient method to remove the dibenzyl group completed the total synthesis of ISJ. The synthetic route of ISJ is shown in Fig. 1b.

First, compound **1** was treated with thionyl chloride, affording compound **2**. A Friedel-Crafts acylation reaction and removal of the 2-methyl group produced the methanone **4** in good yield (65%, over two steps). Converting compound **4** to **5** was conveniently achieved by base-catalyzed cyclization with an 83% yield. Compound **6** was obtained by removing all the methyl groups of compound **5** (refluxing in HBr-HAc, 17 h) with a yield of 97%. Dichlorodiphenylmethane protected the O-hydroxy group of compound **6,** and target compound **7** was obtained with an 85% yield. As the 1-OH of compound **7** can form a hydrogen bond with the adjacent carbonyl group, the propargylic ether, compound **8**, was selectively obtained in the presence of KI and K_2_CO_3_ with a catalytic amount of CuI at a yield of 63%. A p-tosyl group was attached to the free 1-OH of compound **8** to provide the sulfonate, compound **9**, which underwent thermal cyclization to selectively furnish the angular isomer compound **10** in DMF at 150 °C.

Because of its existing olefinic bond, the benzyl group cannot be removed by means of the conventional catalytic hydrogenation method (Pd/C, H_2_). Hydrogenation of the double bond is avoided by refluxing in HAc-H_2_O^35^. However, the yield with this approach is approximately 20%. Therefore, other organic acids were screened in different solvents, including p-toluenesulfonic acid and camphorsulfonic acid (CAS). The optimal reaction was then obtained by refluxing in MeOH-THF (1:1) for 7 h, with a yield of more than 80%. Finally, removing the dibenzyl and p-toluenesulfonyl groups of compound **10** gave the natural product ISJ an overall yield of 19% for ten steps.

### *In vitro* antiproliferative activity

A preliminary screen of ISJ and its structural analogs (compounds **5**–**17**) against HepG2 and QGY-7703 cells was performed at an initial concentration of 50 μM. The results are shown in Table 1. Among these derivatives, compound **7** and ISJ showed greater efficacy than the other compounds against HCC cells. As a result, a second screen was performed, the IC_50_ values of compound **7** and ISJ for HepG2 cells were 7.34 μM and 2.45 μM, respectively; the IC_50_ values of compound **7** and ISJ for QGY-7703 cells were 9.88 μM and 4.65 μM, respectively; the IC_50_ values of compound **7** and ISJ for SMMC-7721 cells were 6.35 μM and 1.63 μM, respectively. However, neither compound showed any marked cytotoxic effects on QSG-7701 hepatocytes with the IC_50_ values of 53.51 μM and 47.67 μM, respectively (Table 2). These findings suggest that compound **7** and ISJ possess selective antiproliferative activities against HCC.

### Effects of compound 7 and ISJ on HCC cell apoptosis

**Figure 2:**
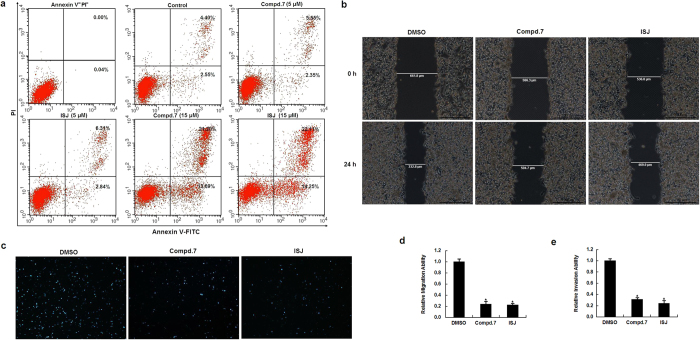
Effects of compound 7 and ISJ on HepG2 cell apoptosis, migration and invasion. (**a**) Flow cytometry analysis using Annexin V-FITC/PI double staining. Compound **7** and ISJ induced apoptosis in HepG2 cells. (**b-c**) Antivascular activities of compound **7** and ISJ. Treatment with compound **7** and ISJ at 10 μM for 24 h resulted in the inhibition of HepG2 migration and invasion. (**b**) Wound healing assay (magnification × 50). (**c**) Matrigel invasion assay (magnification × 100). (**d**) Cell migration was quantified by measuring the distance between the two boundaries of the acellular area, and the result was expressed as a ratio to DMSO-treated cells (control). Data are shown as the mean ± SD of three independent experiments performed in triplicate. *P < 0.05. (**e**) Cell invasion was quantified by counting the mean number of invaded cells (±SD) under the microscope in three randomly selected fields. *P < 0.05.

To further explore the potential mechanisms of the antiproliferative effects induced by compound **7** and ISJ, Annexin V-FITC/PI double staining was performed to quantify cellular apoptosis by flow cytometry. HepG2 cells were cultured for 48 h in the absence or presence of compound **7** or ISJ at different concentrations (5 or 15 μM). Treatment with a low concentration (5 μM) of compound **7** or ISJ did not alter the number of apoptotic cells compared to the control group. In contrast, the percentage of apoptotic cells increased significantly after treatment with higher concentrations of compound **7** or ISJ (15 μM) (Fig. 2a). These results demonstrate that compound **7** and ISJ induce apoptosis in HepG2 cells, which at least partially contributes to their antiproliferative activity.

### Inhibitory effects of compound 7 and ISJ on HCC cell migration and invasion

The effects of compound **7** and ISJ on cell mobility were investigated using wound healing and Matrigel invasion assays. In the wound healing assay, 24-h treatment with 10 μM compound **7** or ISJ strikingly decreased HepG2 cells migration at the edge of the exposed regions by 76.4% and 77.8% (*P* < 0.05), respectively, when compared to the control cultures (Fig. 2b,d). In the invasion assay, the invasivenesses of compound **7**- and ISJ-treated HepG2 cells (24 h, 10 μM) were significantly inhibited by 69% and 76% (*P* < 0.05), respectively, when compared to the HepG2 control cells (Fig. 2c,e). Thus, treatment with compound **7** and ISJ effectively inhibited HepG2 cell migration and invasion.

### Inhibitory effects of compound 7 and ISJ on PKC activity and expression

**Figure 3:**
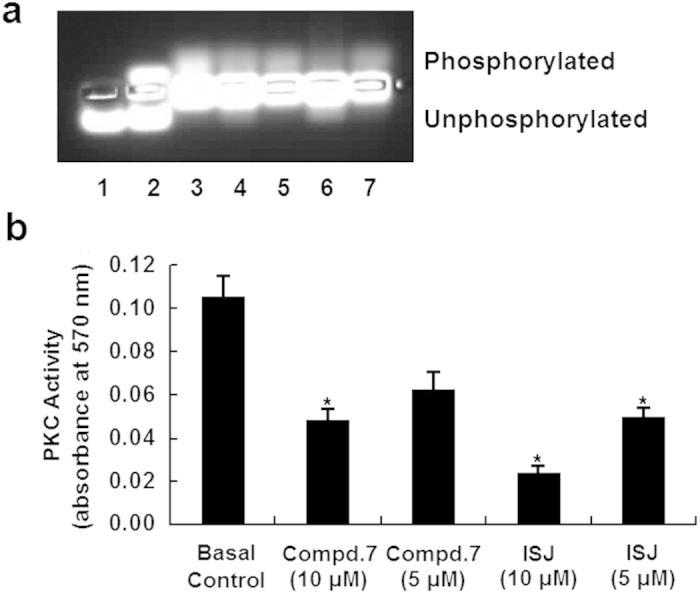
Effects of compound 7 and ISJ on PKC activity in HepG2 cells. The proteins from lysed HepG2 cells were prepared and measured for their ability to phosphorylate a PKC-specific peptide substrate (PLSRTLSVAAK) in a non-radioactive assay. (**a**) Lanes 1 and 2 show the negative (water only) and positive controls (with 20 ng purified PKC). Supernatants from HepG2 cells were subjected to the PKC activity assay in the absence (basal control, lane 3) or presence (lanes 4–7) of the compounds. Compound **7** (lanes 4 and 5) and ISJ (lanes 6 and 7) were added separately at 10 and 5 μM. (**b**) Quantification of (**a**); *P < 0.05 versus the basal control. Data are shown as the mean ± SD of three independent experiments.

The activity of PKC in HepG2 cells was assessed 3 h after the addition of compound **7** or ISJ by measuring the phosphorylation of a PKC-specific target peptide. Compared to the basal control (in the absence of any compound), PKC activity was inhibited by compound **7** and ISJ. Both compound **7** and ISJ displayed greater inhibitory potencies at the higher concentration of 10 μM compared to 5 μM, which was particularly evident for ISJ at 10 μM (Fig. 3a, lane 6). At concentrations of 10 μM and 5 μM, compound **7** inhibited PKC activity by 54.1% and 40.9%, respectively, compared to the basal control; the comparable inhibition rates for ISJ were 77.7% and 52.3%, respectively (Fig. 3b).

**Figure 4:**
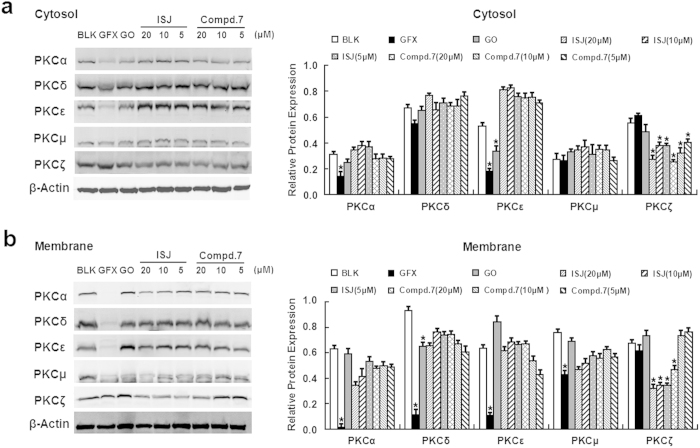
Roles of PKC in HepG2 cells after treatment with GFX, GO, ISJ and compound 7 for 24 h. ISJ and compound **7** selectively inhibited the expression of PKCζ in both the cytosol (**a**) and the plasma membrane (**b**). GFX and GO were used as positive controls at final concentrations of 20 μM. *P < 0.05 versus the BLK controls. Data are shown as the mean ± SD of three independent experiments.

To further determine whether compound **7** and ISJ represent potential candidates for selective PKC expression inhibitors against hepatoma, the expression of PKC isoforms in HepG2 cells was characterized by western blotting. After 24 h of incubation with GFX, GO, ISJ and compound **7** at the indicated concentrations, PKC proteins from the cytosol and membrane of HepG2 cells were evaluated. ISJ and compound **7** treatments reduced both the cytosol and cell membrane expression of PKCζ. Notably, ISJ significantly decreased PKCζ expression in both the cytosolic and membrane fractions (Fig. 4), suggesting that ISJ selectively inhibits aPKC (PKCζ) expression in the cytosol and the translocation of cytosolic aPKC (PKCζ) to the plasma membrane.

### Binding properties of ISJ to PKC isoforms and targeting effects

**Figure 5:**
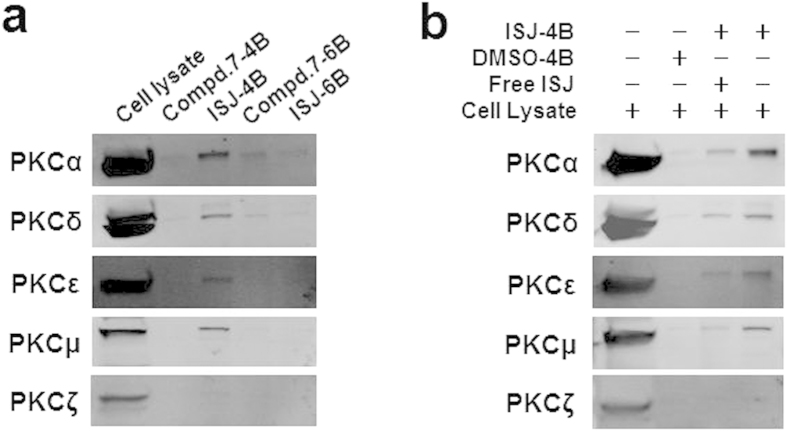
Direct interactions between ISJ and PKC isotypes. (**a**) Immobilized on CNBr-activated Sepharose 4B beads, ISJ directly bound to cPKC (PKCα) and nPKC (PKCδ, PKCε and PKCμ). (**b**) HepG2 cell lysates were incubated with ISJ-conjugated Sepharose 4B beads or with DMSO-conjugated control beads in the absence or presence of free ISJ (20 μM), and the bead-captured proteins were subjected to analysis by immunoblotting.

To identify whether ISJ and compound **7** target PKC in cells, we performed pull-down experiments using ISJ- and compound **7**-coupled Sepharose beads with HepG2 cell lysates. Endogenous cPKC (PKCα) and nPKC (PKCδ, PKCε and PKCμ) in HepG2 lysates were pulled down by ISJ-coupled CNBr-activated Sepharose 4B beads (Fig. 5a). Additionally, the presence of free ISJ (20 μM) resulted in an obvious decline in the amounts of cPKC and nPKC captured by the ISJ-coupled 4B beads (Fig. 5b), indicating that free ISJ competes with conjugated ISJ for binding to cPKC and nPKC, which suggests that the binding is saturated.

**Figure 6:**
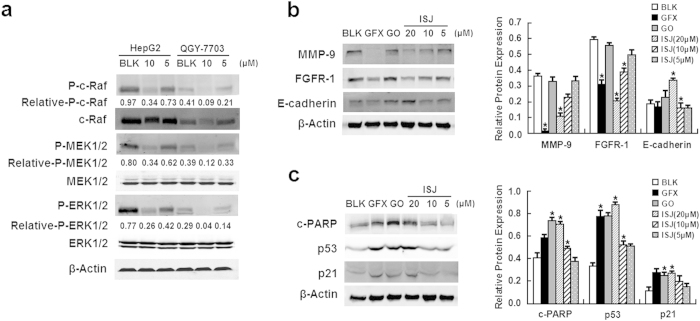
Potencies of ISJ towards the early response of major MAPK phosphorylation (in HepG2 and QGY-7703 cells) and for the late response of decreased invasion and apoptosis (in HepG2 cells). (**a**) ISJ inhibited the phosphorylation of c-Raf, MEK1/2 and ERK1/2 in the MAPK signaling pathway in both HepG2 and QGY-7703 cells. (**b**-**c**) ISJ treatment resulted in decreased invasion (**b**) and apoptosis (**c**) in HepG2 cells. GFX and GO were used as positive controls at final concentrations of 20 μM. *P < 0.05 versus the BLK controls. Data are shown as the mean ± SD of three independent experiments.

Additional PKC targeting effects involved in the early response of major MAPK phosphorylation and the late response of HCC cell invasion and apoptosis were investigated. After 24 h of treatment with ISJ at the indicated concentrations, the total protein lysates from HCC cells were analyzed (Fig. 6). ISJ inhibited the phosphorylated, activated forms of c-Raf, MEK1/2 and ERK1/2 in the MAPK signaling pathway in both HepG2 and QGY-7703 cells (Fig. 6a). Furthermore, the expression levels of matrix metalloproteases 9 (MMP-9) and fibroblast growth factor receptor 1 (FGFR-1) were markedly reduced by ISJ. In contrast, E-cadherin expression was elevated, specifically at the highest concentration of ISJ (20 μM) (Fig. 6b). In addition, we analyzed several representative apoptotic proteins, including cleaved PARP (c-PARP), p53 and p21. The expression levels of these proteins were increased, specifically 20 μM ISJ strongly induced cellular apoptosis (Fig. 6c). These findings support our hypothesis that ISJ restrains HCC cell metastasis and induces HCC cell apoptosis by specifically inhibiting PKC.

### *In vivo* antitumor activities of compound 7 and ISJ

**Figure 7:**
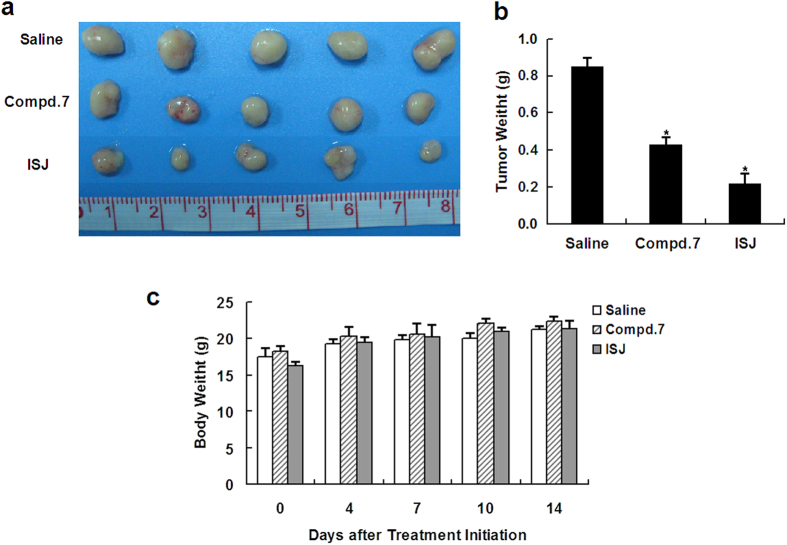
Inhibition of human hepatoma xenograft growth *in vivo* by compound 7 and ISJ. HepG2 cells were transplanted subcutaneously into BALB/c-nu nude mice. Compound **7** or ISJ (5 mg/kg) were given for 14 days. (**a**) Representative tumors from the indicated treatment groups. (**b**) Tumor weight from the indicated treatment groups. (**c**) Body weights were recorded at the indicated days after treatment. *P < 0.05 versus the vehicle-treated controls. Data are shown as the mean ± SD of tumor weight and body weight for five animals per group.

To evaluate the antitumor activities of compound **7** and ISJ *in vivo*, human HCC xenografts were established by subcutaneous injection of HepG2 cells into the backs of nude mice. After the treatment, the body weights of the mice were monitored and recorded at the indicated days. Compared to the vehicle-treated controls, there was no significant change in body weight in the compound **7**- and ISJ-treated groups. Compound **7** and ISJ treatment at a dose of 5 mg/kg significantly inhibited tumor growth over time and reduced the tumor weight by 49.4% and 74.1% (*P* < 0.05), respectively, compared to the controls (Fig. 7).

**Figure 8:**
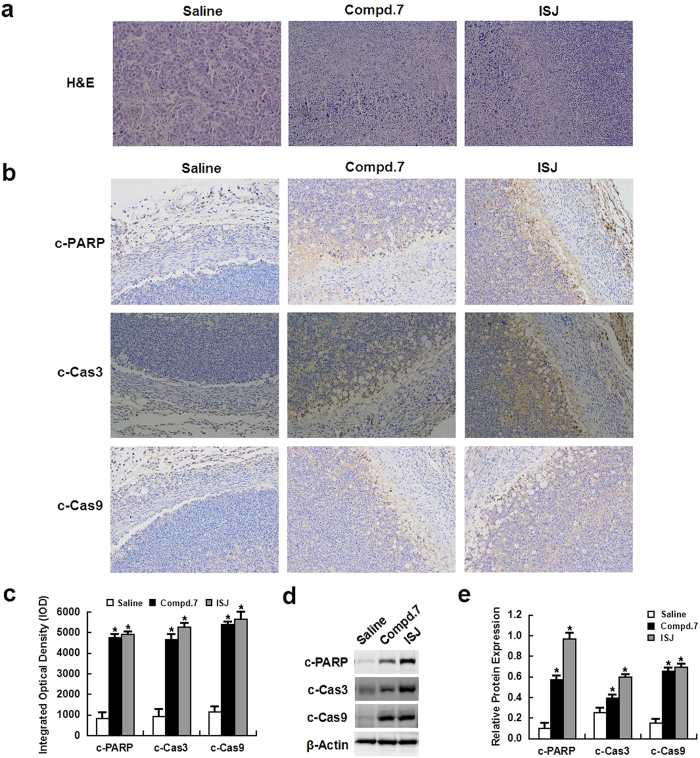
Apoptosis was induced *in vivo* by compound 7 and ISJ (5 mg/kg). (**a**) H&E staining showed significant cell death in tumors from mice treated with compound **7** or ISJ when compared to vehicle-treated controls (magnification × 200). (**b**) IHC indicated strong staining for c-PARP, c-Cas3 or c-Cas9 in tumors from mice treated with compound **7** or ISJ. Vehicle-treated controls showed very weak staining (magnification × 200). (**c**) Quantification of data from (**b**). (**d**) Total protein was extracted from the tumor tissue. The expression of c-PARP, c-Cas3 and c-Cas9 was evaluated by western blotting. (**e**) Quantification of data from (**c**). *P < 0.05 versus the vehicle-treated controls. Data are shown as the mean ± SD of three independent experiments.

At the end of the animal experiment, the control and experimental mice were sacrificed, and the tumors were removed and histologically evaluated by hematoxylin and eosin (H&E) staining and immunohistochemistry (IHC) analysis. Apoptosis was induced by compound **7** and ISJ in xenograft tumors, and H&E staining revealed significant tissue death in compound **7**- and ISJ-treated tumors compared to the tumors from mice treated with the vehicle controls (Fig. 8a). IHC analysis of the tumors revealed upregulated expression of c-PARP, cleaved caspase (c-Cas) 3 and c-Cas9 apoptotic protein levels in the tumors treated with compound **7** and ISJ, whereas the vehicle control tumors showed much weaker staining (Fig. 8b,c). To further validate these *in vivo* results, an immunoblotting assay was used to detect c-PARP, c-Cas3 and c-Cas9 in tumor tissue samples. Compared to the vehicle controls, the levels of these proteins were increased in compound **7**- and ISJ-treated mice, and ISJ was a particularly strong inducer of apoptosis (*P* < 0.05, Fig. 8d,e).

## Discussion

HCC is a leading cause of cancer-related mortality worldwide^36^. Chemotherapy has been an effective method of treating cancers for many years, especially for patients with unresectable tumors. However, the treatment options for HCC are limited, and conventional chemotherapeutic agents, including cyclophosphamide, cisplatin and fluorouracil, have severe side effects^37^. Therefore, the development of new chemotherapeutic drugs is of great significance. Plant-derived natural products and synthetic analogs are significant components of cancer chemotherapy and have been shown to suppress metastasis and induce apoptosis in malignant tumor cells while exerting low toxicity to normal cells and minimal adverse effects^38^,^39^.

In our previous studies, a new series of derivatives of phenyl-substituted polyoxygenated xanthone were synthesized and evaluated for *in vitro* cytotoxicity against HCC cells and hepatocytes^33^,^34^. In the present study, ISJ and compound **7** demonstrated potency at several cellular-level activities, thereby supporting the derivatives’ antitumor effects. First, the natural product ISJ and its intermediate, compound **7**, exhibited more efficacious antiproliferative activities for HCC cells and exerted no apparent toxicity to hepatocytes. Second, ISJ and compound **7** effectively induced HepG2 cell apoptosis, and third, they significantly suppressed the migration and invasion of HepG2 cells.

The PKC family of serine/threonine protein kinases contains multiple isoforms. PKC overexpression is correlated with the progression and prognosis of malignant diseases, including HCC^11^,^12^,^13^. To begin to address the regulatory mechanisms of ISJ and compound **7** on PKC, we examined their inhibitory effects using an *in vitro* kinase assay and found that ISJ exhibited stronger PKC inhibitory activity compared to compound **7**. Different PKC isotypes are known to play distinct regulatory roles. Therefore, immunoblotting was conducted to characterize the expression of PKC isoforms in HepG2 cells and explore whether ISJ and compound **7** represent potential candidates for selective PKC expression inhibitors in hepatoma. Increased PKCζ expression in different types of human cancers supports the clinical correlation of PKCζ as a tumor promoter, with high PKCζ activity predicting poor survival in several cancers^40^,^41^,^42^. Recently, an anti-apoptotic effect was attributed to PKCζ^43^, and PKCζ inhibition reduced tumor cell proliferation and increased pancreatic tumor necrosis^44^. Furthermore, specific inhibition of PKCζ through RNAi impaired breast cancer and glioblastoma cell invasion *in vitro*^45^ and reduced prostate cancer cell invasion both *in vitro* and *in vivo*^46^. We observed a significant downregulation of PKCζ expression in the cytosolic and membrane fractions when HepG2 cells were exposed to ISJ. Therefore, ISJ likely acts as a selective inhibitor of PKC expression and further direct interaction investigations suggest that ISJ possess differential actions against different PKC isotypes. ISJ selectively inhibited the expression of aPKC (PKCζ) in the cytosol and the translocation of cytosolic aPKC (PKCζ) to the plasma membrane. Indeed, the translocation of PKC in response to agonists is an indicator of enzyme activation^47^. We also observed direct interactions between ISJ and cPKC (PKCα) or nPKC (PKCδ, PKCε and PKCμ).

The cascades downstream of PKC include Raf/MEK/ERK MAPK signaling pathways. The epithelial-to-mesenchymal transition (EMT), in which cells undergo transformation from an epithelial phenotype to a motile and invasive phenotype, is a critical step in the metastatic dissemination of malignant cells^48^,^49^,^50^. This phenomenon is connected with increased MMP activity and loss of E-cadherin expression^51^. Matrix degradation by MMP-9 is dependent on PKC activity^52^,^53^, and E-cadherin-mediated cell-cell adhesion plays important roles in cell polarity maintenance^54^. In HCC, the expression of E-cadherin is significantly downregulated, which is associated with tumor metastasis and prognosis^53^ and the functional overexpression of E-cadherin in HCC cells significantly inhibits cell invasion^55^,^56^. In addition, FGFR-1 overexpression increases HCC cell proliferation and invasion, thereby facilitating the progression of HCC^57^,^58^. We investigated the PKC targeting effects of ISJ, specifically by examining proteins involved in the MAPK signaling pathways and those associated with invasion and apoptosis. Our results showed that ISJ inhibited the early response of major MAPK phosphorylation and the late response of HCC cell invasion and proliferation. Furthermore, in the hepatoma xenograft model, ISJ pretreatment showed potent antihepatoma activity *in vivo*.

Taken together, our current findings indicate that ISJ, the lead polyoxygenated xanthone-based PKC inhibitor, represents a novel and promising antitumor drug to target HCC.

## Materials and Methods

### Materials

ISJ and its intermediate, compound **7**, were synthesized in our laboratory. GF 109203X (GFX) and Go 6976 (GO) were purchased from Tocris Bioscience (Ellisville, MO). 5-Fluorouracil (5-Fu) was obtained from Sigma (St. Louis, Mo). Primary antibodies were purchased from Cell Signaling Technology (Beverly, MA), including those specific for β-actin (#4970), MMP-9 (#2270), E-Cadherin (#3195), FGFR-1 (#9740), PKCε (#2683), c-Raf (#9422), MEK1/2 (#9122), ERK1/2 (#9102), Phospho-c-Raf (#9427), Phospho-MEK1/2 (#9154), and Phospho-ERK1/2 (#4370). The PKC Isoform Antibody Sampler Kit (#9960) and the Apoptosis Antibody Sampler Kit (#9915) were also purchased from Cell Signaling Technology. Antibodies to p53 (AP062) and p21 (AP021) were purchased from Beyotime (Shanghai, China).

### Compound 7 and ISJ synthesis

The final compound ISJ, and its intermediate, compound **7**, were synthesized as described in Fig. 1 and as detailed in the Supplementary material 1.

### Cell culture

The human HCC cell lines QGY-7703, SMMC-7721 and HepG2 and the liver cell line QSG-7701 were supplied by the Cell Resource Center, Institute of Life Sciences, Chinese Academy of Medical Science. QGY-7703, SMMC-7721 and QSG-7701 cells were cultured in 1640 medium (Gibco), and HepG2 cells were cultured in DMEM medium (Gibco) at 37 °C in moist air supplemented with 5% CO_2_. All media were supplemented with 10% fetal bovine serum (FBS) and 100 Units/mL of penicillin/streptomycin.

### Cell proliferation assay

The cell viability assay was performed as previously reported using a Cell Counting Kit-8 (CCK-8; Dojindo, Kumamoto, Japan)^33^,^34^. The relative cell viability was presented as % inhibition and calculated using the following formula:


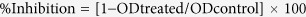


### Apoptosis assay

HepG2 cells were seeded in six-well plates (5 × 10^5^ cells/mL) for 24 h and then treated with various amounts of the compounds or 1% DMSO (as control). After 48 h, the cells floating in the medium were collected, and the adherent cells were detached with 0.05% trypsin, washed twice with cold PBS, and centrifuged at 1000 rpm for 5 min at 4 °C. Subsequently, HepG2 cells were gently resuspended in the binding buffer. Thereafter, the cells were stained using the Annexin V-FITC/PI apoptosis detection kit (BD Biosciences, San Jose, CA, USA). After incubation at room temperature for 15 min in the dark, the apoptotic cells were immediately analyzed by flow cytometry (FACS Calibur).

### Migration and invasion assay

Cell migration and invasion potencies were studied using wound healing and Matrigel invasion assays. The wound healing assay was performed by scratching the cell surface with a 10 μL pipette tip. Then, the cells were treated with compound **7** and ISJ (10 μM) and incubated for 24 h before being photographed under a microscope (DMI3000 B, Leica). Wound closure was evaluated using ImageJ software. Invasion through a Matrigel-coated filter was measured in transwell chambers (Corning Costa) as described previously^59^, with minor modifications. Briefly, HepG2 Cells (2 × 10^5^/well) were suspended in serum-free medium and added to the upper chamber of the Matrigel plate. FBS (10%) was used as the chemoattractant in the lower chamber. After incubation for 24 h, the non-invading cells were removed from the upper surface of the insert membrane with a cotton swab. The invading cells were fixed with methanol and stained with Giemsa (Sigma). Cells in five randomly selected fields were photographed, and the numbers of cells that crossed the membrane were counted.

### PKC activity assay

The PKC activity assay was carried out as previously reported^33^, according to the instructions of the PepTag non-radioactive PKC assay kit (Promega)^60^. The bands were photographed using the Syngene GBOX-iCHEMI-XR system, and the phosphorylated bands were cut out for quantification measurement at 570 nm.

### Cell-based pull-down assay

Compound **7** and ISJ were coupled to CNBr-activated Sepharose 4B beads and Epoxy-activated Sepharose 6B beads (GE Healthcare) separately, according to the manufacturer’s suggested protocol. The control beads were prepared by incubating the beads with DMSO. Proteins (500 μg) from lysed HepG2 cells were mixed with either the control beads, compound **7**-coupled beads, or ISJ-coupled beads (100 μL) in reaction buffer [50 mM Tris (pH 7.5), 5 mM EDTA, 150 mM NaCl, 1 mM DTT, 0.01% NP-40, 2 μg/mL BSA, 1 mM PMSF, 1% protease inhibitor mixture]. After incubation with gentle rocking overnight at 4 °C, the beads were washed 5 times with washing buffer [50 mM Tris (pH 7.5), 5 mM EDTA, 150 mM NaCl, 1 mM DTT, 0.01% NP-40, 1 mM PMSF], and binding was visualized by western blotting.

### Western blot

The total membrane and cytosolic proteins were extracted using a Membrane and Cytosol Protein Extraction Kit (Beyotime). Extracted proteins from cells or animal tissues were separated by SDS-PAGE and transferred to polyvinylidene difluoride (PVDF) membranes (Bio-Rad). The PVDF membranes were blocked with 5% nonfat milk and incubated with primary antibodies overnight, followed by incubation with a donkey anti-rabbit or anti-mouse secondary antibody (IRDye 800, LI-COR, Biosciences). The images were captured using the Odyssey Infrared Imaging System (LI-COR, Biosciences), according to the manufacturer's instructions. The intensity was quantitative with Quantity One software (Bio-Rad). To ensure equal protein loading, each membrane was stripped and reprobed with an anti-β-actin antibody. All experiments were repeated independently no less than three times.

### Antitumor activity *in vivo*

Four-week-old male BALB/c-nu nude mice (15–20 g) were obtained from the Shanghai SLAC Laboratory Animal Co., Ltd. (Shanghai, China). The animals were maintained under specific pathogen-free conditions with food and water supplied *ad libitum* in the Laboratory Animal Center of the Second Military Medical University. All animal experiments were carried out in accordance with the Guide for the Care and Use of Laboratory Animals of the National Institutes of Health and were approved by the Committee on the Ethics of Animal Experiments of the Second Military Medical University, China.

HepG2 cells were harvested and resuspended in PBS. A total of 5 × 10^6^ cells were subcutaneously injected into the right flank. Tumor volume was calculated using the following formula: V = (L × W^2^)/2, where *L* is the length and *W* is the width of the tumor nodules measured with vernier calipers. Once the volume of the tumors reached 75–100 mm^3^, the mice were randomly divided into three groups (n = 5). The mice were treated every other day for two weeks with tail vein injections of either vehicle (5% ethanol and 2.5% polyethylene glycol in saline), compound **7**, or ISJ (5 mg/kg in the vehicle). Body weights were measured before each drug injection. After the 15th day, the mice were euthanized, and the tumors were isolated, weighed and photographed.

To further confirm the antitumor activities of compound **7** and ISJ *in vivo*, histological analysis was performed on the hepatoma xenografts. Briefly, the samples were fixed in a 10% formalin solution, processed, embedded, sectioned and either H&E stained to reveal tumor tissue necrosis or IHC stained using antibodies against c-PARP, c-Cas3 or c-Cas9. The H&E and IHC images were obtained on a Leica DMI3000 B phase-contrast microscopy. The immunoreactive areas in the IHC images were quantified using ImagePro Plus 6.0 software (Media Cybernetics, Silver Spring, MD). The integrated optical density (IOD) values were represented as the mean ± SEM.

### Statistical analysis

All experiments were performed in triplicate and repeated at least twice, with representative experiments selected for figures. The intergroup data were compared using Student’s t-test, and multivariate analysis was determined by one-way analysis of variance (ANOVA). SPSS software (version 16.0) was used for statistical tests with a minimal level of significance at *P* < 0.05.

## Additional Information

**How to cite this article**: Yuan, X. *et al.* Inhibition of protein kinase C by isojacareubin suppresses hepatocellular carcinoma metastasis and induces apoptosis *in vitro* and *in vivo*. *Sci. Rep.* **5**, 12889; doi: 10.1038/srep12889 (2015).

## Supplementary Material

Supplementary Information

## Figures and Tables

**Table 1:** Inhibition rates of compounds 5–17 and ISJ against human HepG2 and QGY-7703 HCC cells at 50 μM.

Compd.^a^	Inhibitory Ratio (%)	
	HepG2	QGY-7703
**5**	22.61	20.81
**6**	13.80	−0.66
**7**	90.99	79.93
**8**	6.88	−9.85
**9**	12.46	−3.98
**10**	18.01	0.66
**11**	45.36	21.44
**12**	26.12	20.97
**13**	47.29	16.17
**14**	25.40	13.15
**15**	24.07	−1.75
**16**	15.95	13.26
**17**	17.52	0.99
**ISJ**	91.54	83.69

^a^Chemical structures of compounds **12**–**17** are shown in the Supplementary material 2.

**Table 2:** Antiproliferative activities of target compounds against human HCC cells and hepatocytes.

Compd.	IC_50_^b^ (μM)			
	HepG2	QGY-7703	SMMC-7721	QSG-7701
**7**	7.34 ± 0.09	9.88 ± 0.18	6.35 ± 0.42	53.51 ± 0.13
ISJ	2.45 ± 0.23	4.65 ± 0.32	1.63 ± 0.05	47.67 ± 0.08
5-Fu	12.48 ± 0.11	15.66 ± 0.07	14.55 ± 0.36	0.60 ± 0.03

^b^Data are expressed as the mean ± SD from the dose-response curves of at least three independent experiments.
