# Supplementary material for: Inhibition of protein kinase C by isojacareubin suppresses hepatocellular carcinoma metastasis and induces apoptosis in vitro and in vivo
Source: Sci Rep. 2015 Aug 6;5:12889. doi: 10.1038/srep12889 (PMC4526861; doi:10.1038/srep12889)

Inhibition of protein kinase C by isojacareubin suppresses hepatocellular carcinoma metastasis and induces apoptosis in vitro and in vivo

Xing Yuan[1,#], Hao Chen[1,#], Xia Li[1], Ming Dai[1], Huawu Zeng[1], Lei Shan[1,*], Qingyan Sun[2,*], Weidong Zhang[1,3,*]

Supplementary material 1

Synthetic method for compound 7 and ISJ

General Procedures for Chemistry

Chemicals were purchased from commercial sources and were used without further purification unless otherwise noted. Nuclear magnetic resonance (NMR) spectra were recorded using TMS as the internal standard in DMSO with a Bruker BioSpin GmbH spectrometer at 300 MHz or 600 MHz. When peak multiplicities are reported, the following abbreviations are used: s = singlet, d = doublet, t = triplet, m = multiplet, and dd = doublet of doublets; ESI-MS was recorded on an Agilent 1100 LC/MSD (70 eV) spectrometer. High resolution mass spectra (HRMS) were recorded on a Waters Q-Tof micro. Melting points (mp) were determined using a microscope melting point apparatus with an automatic temperature control system (XT4A). Flash column chromatography was performed using silica gel (200-300 mesh).

1,3,5,6-tetramethoxy-9H-xanthen-9-one (5): To a solution of compound **1** (2.78 g, 13.1 mmol) in dry benzene (60 mL) was added $SOCl_2$ (5 mL) in one portion. The mixture was warmed up to 80 ℃ for 4 h under stirring. After cooling to room temperature, the organic solvent was removed in vacuum to get crude compound **2** for next step without further purification. Then it was dissolved in dry ether (80 mL) with 1,3,5-trimethoxybenzene (2.20 g, 13.0 mmol) was added dry $AlCl_3$ (5.0 g, 37.88 mmol) in portions under stirring at

room temperature. After 24 h, the mixture was poured into 10% HCl (aq), then was extracted with CH_2Cl_2.The organic layer was washed with brine, dried over Na_2SO_4, vacuumed and evaporated to yield a product, which was purified by column chromatography to afford white solid compound **4** (2.97 g, 8.5 mmol, 65%). To a solution of compound **4** (2.97 g, 8.5 mmol) and 10% Bu_4N•OH (45 mL) in pyridine (100 mL) and water (50 mL) was refluxed at 110 ℃ for 36 h. After cooling to room temperature, the mixture was poured into ice water, and then acidified with 10% HCl. After extraction with EtOAc, the organic layer was dried over Na_2SO_4 and concentrated under vacuum to get crude product, which was purified by column chromatography to afford compound **5** (2.24 g, 7.08 mmol, 83%); white solid; mp 138-140 ℃; ^{1}H NMR (300 MHz, DMSO-d_6) δ = 7.78 (d, J = 9.0 Hz, 1H), 7.15 (d, J = 9.1 Hz, 1H), 6.71 (d, J = 2.4 Hz, 1H), 6.49 (d, J = 2.4 Hz, 1H), 3.92 (s, 3H), 3.91 (s, 3H), 3.89 (s, 3H), 3.85 (s, 3H); ^{13}C NMR (75 MHz, DMSO-d_6) δ = 174.9, 166.5, 163.3, 161.0, 158.3, 150.4, 137.3, 123.0, 119.0, 111.0, 107.7, 97.4, 95.2, 62.8, 58.2, 58.0, 57.9; HR-ESI-MS calcd for $C_{17}H_{16}O_6$ 316.0947 $[M+H]^+$, found 316.0949.

1,3,5,6-tetrahydroxy-9H-xanthen-9-one (6): To a solution of compound **5** (7.71 g, 21.0 mmol) in HBr (50 mL) and HAc (50 mL) was refluxed in oil, then HBr (10 mL) and HAc (10 mL) were added every one hour. After 17 h, the starting material was consumed, and then concentrated to 200 mL under vacuum. After cooling to room temperature, the mixture was poured into water (600 mL), then filtered to get crude product, recrystallized (in methanol) to get

compound **6** (97.5%); yellow solid; mp > 300 ℃; ^1H NMR (300 MHz, DMSO-d_6) δ = 6.20 (d, J = 2.5 Hz, 1H), 6.46 (d, J = 2.5 Hz, 1H), 6.91 (d, J = 8.5 Hz, 1H), 7.57 (d, J = 8.5 Hz, 1H), 11.49, 12.55 (s, 2H, 1, 3-OH); ^{13}C NMR (75 MHz, DMSO-d_6) δ = 179.7, 165.1, 162.9, 157.4, 151.9, 146.1, 132.5, 115.9, 113.1(2C), 101.5, 97.9, 94.0; HR-ESI-MS calcd for $C_{13}H_8O_6$ 261.0394 $[M+H]^+$, found 261.0387.

7,9-dihydroxy-2,2-diphenyl-6H-[1,3]dioxolane[4,5-c]xanthen-6h-one (7): To a solution of compound **6** (0.13 g, 0.5 mmol) in Ph_2O (10 mL) and Ph_2CCl_2 (0.18 g, 0.75 mmol) was stirred for 30 min at 175 ℃ under argon. After cooling to room temperature, petroleum ether (100 mL) was added to the reaction mixture, then left overnight, filtered to get crude product, which was purified by column chromatography (PE/EA=8:1) to afford compound **7** (0.18 g, 85%); pale yellow; mp: 212-214 ℃; ^1H NMR (500 MHz, DMSO-d_6) δ = 12.86 (s, 1H), 11.07 (s, 1H), 7.72 (dt, J = 8.6, 1.6 Hz, 1H), 7.59-7.53 (m, 4H), 7.45 (dd, J = 5.4, 1.9 Hz, 6H), 7.19 (dt, J = 8.4, 2.0 Hz, 1H), 6.39 (t, J = 1.8 Hz, 1H), 6.18 (d, J = 2.1 Hz, 1H); ^{13}C NMR (125MHz, DMSO-d_6) δ = 178.8, 165.7, 163.1, 156.9, 152.0, 139.9, 138.5, 132.8, 129.8, 128.7 (4C), 125.9 (6C), 120.6, 119.5, 116.2, 106.4, 101.6, 98.4, 94.3; HR-ESI-MS calcd for $C_{26}H_{16}O_6$ 425.1020 $[M+H]^+$, found 425.1021.

7-hydroxy-9-(2-methylbut-3-yn-2-yloxy)-2,2-diphenyl-6H[1,3]dioxolo[4,5-c] xanthen-6-one (8): To a solution of compound **7** (619 mg, 1.46 mmol), KI (400 mg, 2.41 mmol), K_2CO_3 (220 mg, 1.59 mmol) and CuI (14.0 mg, 73.8 μmol) in

dry acetone (8 mL) was added 2-chloro-2-methyl-1-butyne(164 mg, 1.61 mmol) with stirring under argon. The reaction mixture was stirred at 45 ℃ for 2 h and then cooled to room temperature. The mixture was acidified to PH=5-6 and then was extracted with EtOAc, washed with water, dried over Na_2SO_4, concentrated to get crude product, which was purified by column chromatography (PE/EA=4:1) to afford compound **8** (453 mg, 63%); yellow solid; ^{1}H NMR (300 MHz, DMSO-d_6) δ = 7.79 (d, *J* = 8.5 Hz, 1H), 7.64-7.55 (m, 4H), 7.52-7.44 (m, 6H), 7.27 (d, *J* = 8.4 Hz, 1H), 6.87 (d, *J* = 2.1 Hz, 1H), 6.55 (d, *J* = 2.1 Hz, 1H), 3.93 (s, 1H), 1.70 (s, 6H); ^{13}C NMR (75 MHz, DMSO-d_6) δ = 178.8, 165.2, 163.1, 156.9, 152.0, 139.9, 138.5, 132.8, 129.8, 128.7 (4C), 125.9 (6C), 120.6, 119.5, 116.2, 106.4, 101.6, 98.4, 94.3; HR-ESI-MS calcd for $C_{31}H_{22}O_6$ 491.1489 $[M+H]^+$, found 491.1497.

10,10-dimethyl-6-oxo-2,2-diphenyl-6,10-dihydro-[1,3]dioxolo[4,5-c]pyrano[3,2-h]xanthen-7-yl 4-methylbenzenesulfonate (10): To a solution of compound **8** (617 mg, 1.26 mmol), TsCl (777 mg，4.08 mmol) and K_2CO_3 (777 mg, 4.08 mmol) in acetone was refluxed for 90 min. After cooling to room temperature, the mixture was filtered through a Celite pad, and the filtrate was concentrated to give the crude product, which was purified by column chromatography (PE/EA=4:1) to afford white solid compound **9** (673 mg, 83%). A round-bottom flask was charged with compound **9** (296 mg, 0.46 mmol) in DMF (20 mL), and the mixture was refluxed for 2 h. After cooling to room temperature, the mixture was concentrated to afford the crude product, which

was purified by column chromatography to afford compound **10** (252 mg, 85%); yellow solid; ^{1}H NMR (600 MHz, DMSO-d_6) δ = 7.79 (d, *J* = 8.3 Hz, 2H), 7.64 (d, *J* = 8.5 Hz, 1H), 7.60 (dt, *J* = 4.5, 2.5 Hz, 4H), 7.48 (dd, *J* = 7.2, 4.2 Hz, 7H), 7.41 (d, *J* = 8.2 Hz, 2H), 7.21 (d, *J* = 8.5 Hz, 1H), 6.86 (d, *J* = 10.1 Hz, 1H), 6.40 (s, 1H), 6.00 (d, *J* = 10.1 Hz, 1H), 2.34 (s, 3H), 1.46 (s, 6H); ^{13}C NMR (150 MHz, DMSO-d_6) δ = 174.2, 160.1, 157.0, 154.4, 151.6, 145.7, 144.7, 141.8 (2C), 134.8, 132.7, 130.1 (2C), 128.5 (2C), 128.2 (4C), 127.7 (6C), 119.0, 116.9, 116.3, 111.2, 110.1, 108.1, 102.8, 84.5, 75.1, 72.7, 29.5 (2C), 21.5; HR-ESI-MS calcd for $C_{38}H_{28}O_8S$ 644.1505 [M+H]$^+$, found 644.1567.

10,11-dihydroxy-3,3-dimethyl-7-oxo-3,7-dihydropyrano[2,3-c]xanthen-6-yl 4-methylbenzenesulfonate (11): A round-bottom flask was charged with compound **10** (644 mg, 1.0 mmol) and CAS (300 mg) in MeOH (20 mL) and THF (20 mL), and the mixture was refluxed at 55 ℃ for 2 h. After cooling to room temperature, the mixture was concentrated and extracted with EtOAc and sat. $NaHCO_3$. The organic layer was washed with sat. NaCl, dried over Na_2SO_4 and concentrated to afford the crude product, which was purified by column chromatography to afford compound **11** (441 mg, 92%); yellow solid; ^{1}H NMR (600 MHz, DMSO-d_6) δ = 7.78 (d, *J* = 8.3 Hz, 2H), 7.40 (d, *J* = 8.1 Hz, 2H), 7.37 (d, *J* = 8.7 Hz, 1H), 7.11 (d, *J* = 10.0 Hz, 1H), 6.87 (d, *J* = 8.7 Hz, 1H), 6.35 (s, 1H), 5.94 (d, *J* = 10.2 Hz, 1H), 2.34 (s, 3H), 1.43 (s, 6H); ^{13}C NMR (150MHz, DMSO-d_6) δ = 158.0, 154.0, 153.3, 149.0, 147.5, 146.9, 134.0, 133.8, 132.7, 131.7, 130.4, 118.4, 116.9, 116.5, 115.0, 111.1, 110.5, 109.6,

80.5, 61.6, 29.5, 23.0, 15.9; HR-ESI-MS calcd for $C_{25}H_{20}O_8S$ 481.0952 $[M+H]^+$, found 481.0941.

<u>6,10,11-trihydroxy-3,3-dimethylpyrano[2,3-c]xanthen-7(3H)-one (ISJ):</u> To a solution of compound **11** (80 mg, 0.17 mmol) in 3% KOH (600mg KOH in 10 mL H_2O and 10 mL EtOH) (5 mL) was refluxed at 100 ℃ and added 3% KOH (5 mL) every 15 min. After 1 h, the reaction mixture was cooled to room temperature and adjusted to pH=7 with 10% HCl. After the EtOH was evaporated, the mixture was extracted with EtOAc, dried over Na_2SO_4 and concentrated to afford the crude product, which was purified by column chromatography to afford ISJ (41 mg, 72.7%); yellow solid; ^{1}H NMR (600 MHz, DMSO-d_6) δ = 13.26 (s, 1H), 10.79 - 9.27 (m, 2H), 7.50 (d, *J* = 8.5 Hz, 1H), 7.06 (d, *J* = 10.1 Hz, 1H), 6.92 (d, *J* = 8.6 Hz, 1H), 6.17 (s, 1H), 5.76 (d, *J* = 10.0 Hz, 1H), 1.42 (s, 6H); ^{13}C NMR (150 MHz, DMSO-d_6) δ = 181.8, 164.2, 161.5, 154.4, 153.3, 147.9, 134.3, 129.1, 118.0, 116.8, 115.3, 114.7, 104.2, 102.7, 100.2, 80.0, 29.7 (2C); HR-ESI-MS calcd for $C_{18}H_{14}O_6$ 327.0863 $[M+H]^+$, found 327.0847.

Figure S1. The ^{1}H NMR and ^{13}C NMR spectrums of compound **7** in DMSO and HRMS

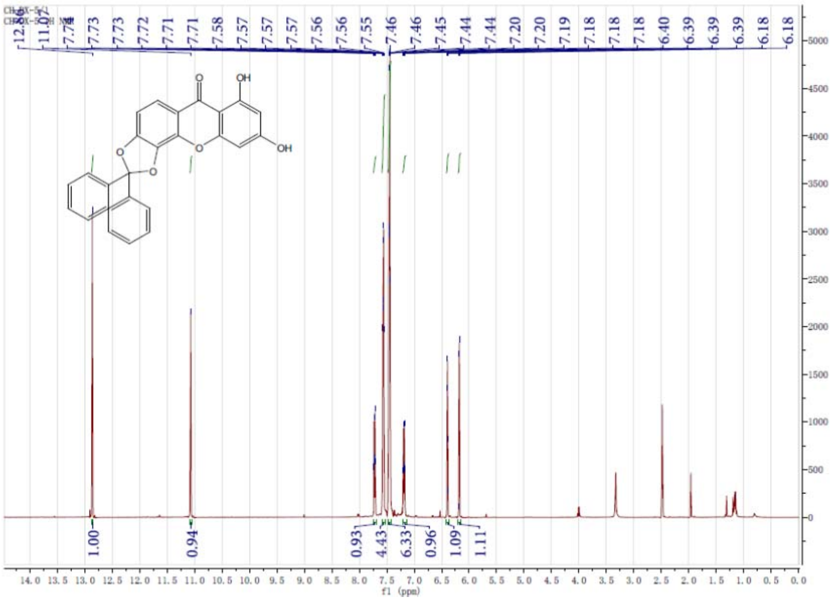

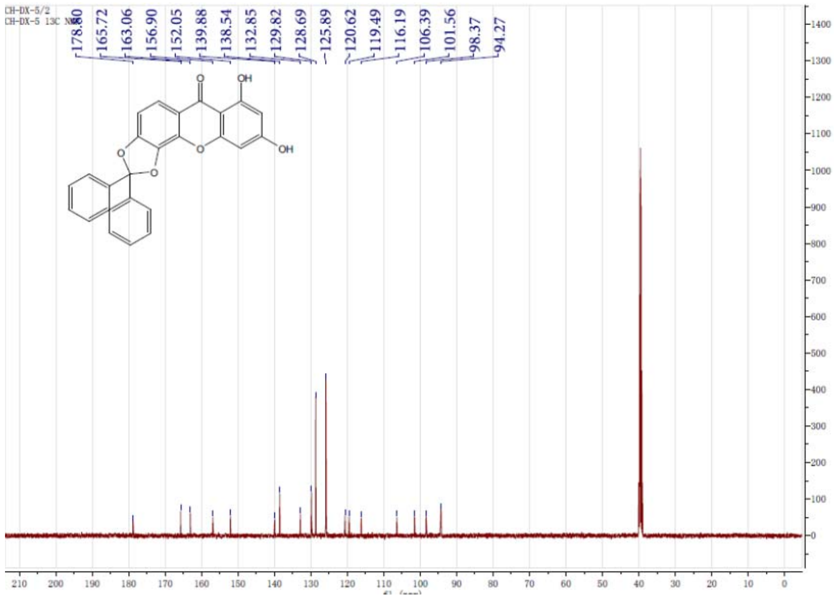

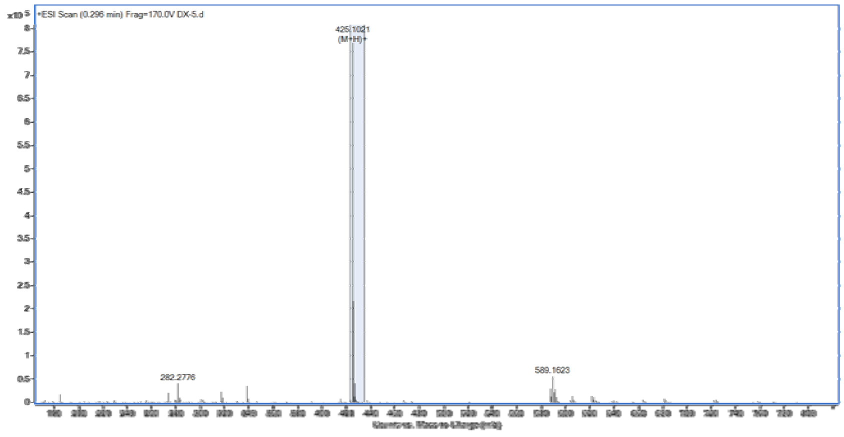

Figure S2. The ^{1}H NMR and ^{13}C NMR spectrums of ISJ in DMSO and HRMS

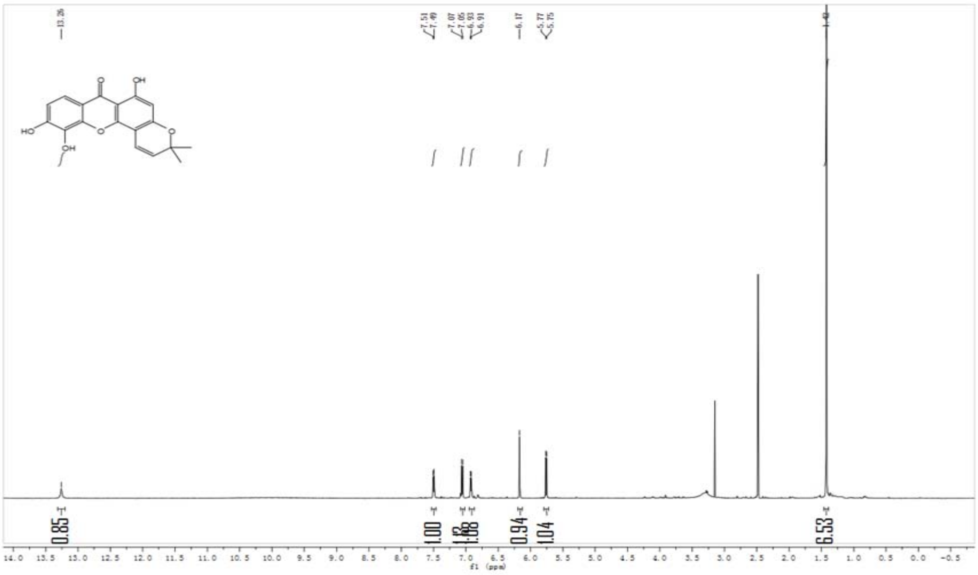

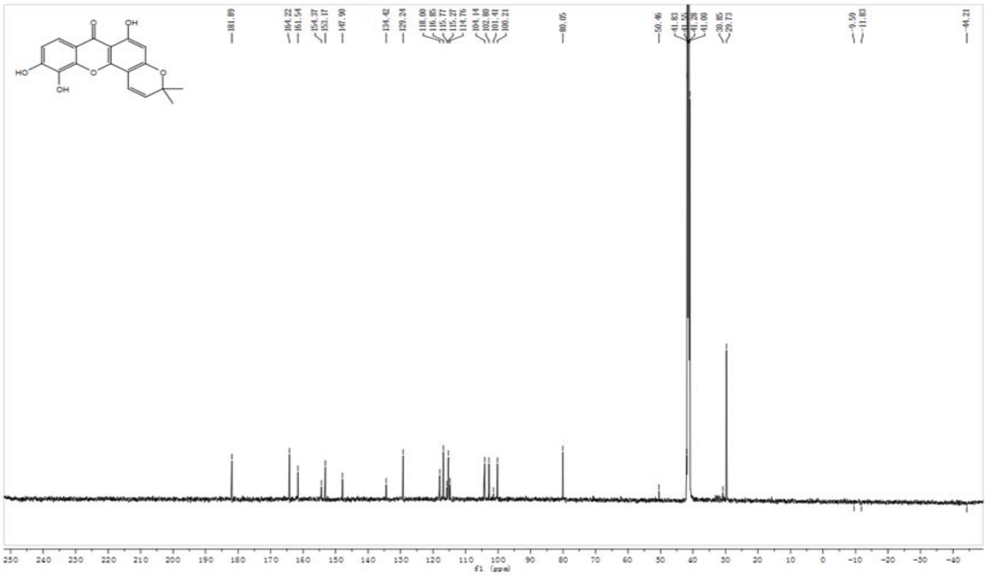

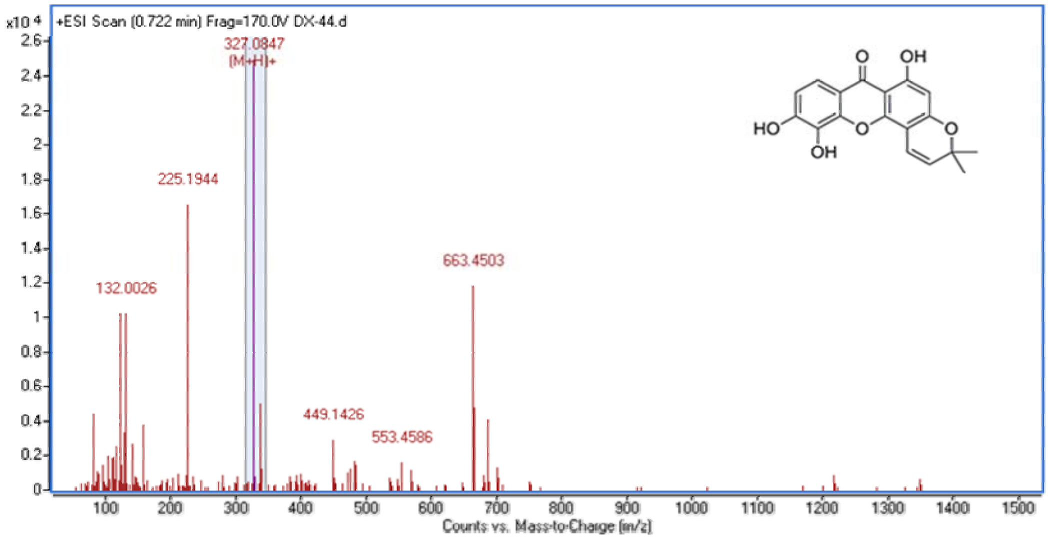

Supplementary material 2

Chemical structures of ISJ structural analogs 12-17

- 1,3,5,6-tetramethoxy-9-oxo-9H-xanthene-4-carbaldehyde (**12**):

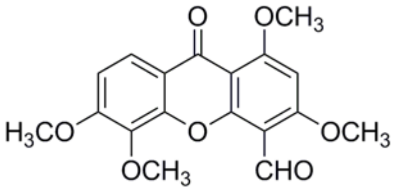

- (E)-ethyl-3-(1,3,5,6-tetramethoxy-9-oxo-9H-xanthen-4-yl)acrylate (**13**):

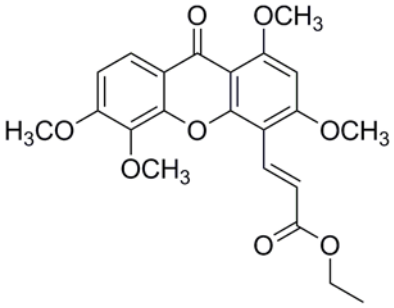

- (2S,3R)-ethyl 2,3-dihydroxy-3-(1,3,5,6-tetramethoxy-9-oxo-9H-xanthen-4-yl) propanoate (**14**):

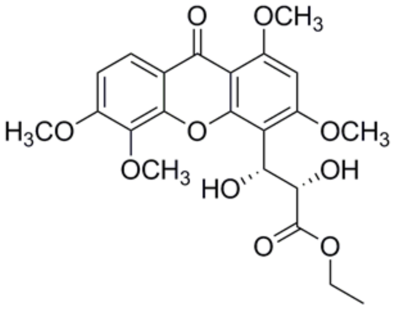

- 4-iodo-1,3,5,6-tetramethoxy-9H-xanthen-9-one (**15**):

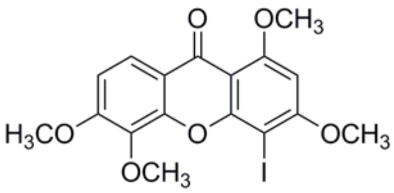

- (E)-4-(3-hydroxy-3-methylbut-1-enyl)-1,3,5,6-tetramethoxy-9H-xanthen-9-one (**16**):

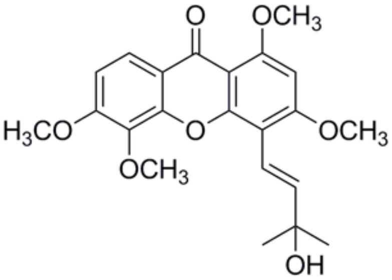

- 1,3,5,6-tetramethoxy-4-((1S,2R)-1,2,3-trihydroxy-3-methylbutyl)-9H-xanthen-9-one (**17**):

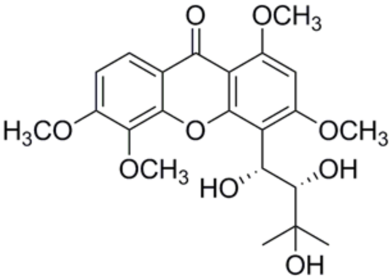

Supplement: Supplementary Information [file srep12889-s1.pdf]
